# The role of rapid maxillary expansion in the promotion of oral and general health

**DOI:** 10.1186/s40510-015-0105-x

**Published:** 2015-10-07

**Authors:** James A. McNamara, Roberta Lione, Lorenzo Franchi, Fernanda Angelieri, Lucia HS Cevidanes, M. Ali Darendeliler, Paola Cozza

**Affiliations:** Department of Orthodontics and Pediatric Dentistry, School of Dentistry, The University of Michigan, Ann Arbor, MI USA; Cell and Developmental Biology, School of Medicine, The University of Michigan, Ann Arbor, MI USA; Center for Human Growth and Development, The University of Michigan, Ann Arbor, MI USA; Department of Clinical Sciences and Translational Medicine, University of Rome “Tor Vergata”, Rome, Italy; Department of Surgery and Translational Medicine, University of Florence, Via del Ponte di Mezzo, 46-48, Florence, 50127 Italy; Department of Orthodontics, São Paulo Methodist University, São Bernardo do Campo, Brazil; Discipline of Orthodontics, Faculty of Dentistry, University of Sydney, Sydney, Australia; Sydney Dental Hospital, Sydney South West Area Health Service, Sydney, Australia; Department of Dentistry, UNSBC, Tirana, Albania

**Keywords:** Dentofacial orthopedics, Rapid maxillary expansion, Oral health, General health, OSAS, Breathing disorders, Muscle activity

## Abstract

Rapid maxillary expansion (RME) is an effective orthopedic procedure that can be used to address problems concerned with the growth of the midface. This procedure also may produce positive side effects on the general health of the patient. The aim of the present consensus paper was to identify and evaluate studies on the changes in airway dimensions and muscular function produced by RME in growing patients. A total of 331 references were retrieved from a database search (PubMed). The widening of the nasal cavity base after midpalatal suture opening in growing patients allows the reduction in nasal airway resistance with an improvement of the respiratory pattern. The effects of RME on the upper airway, however, have been described as limited and local, and these effects become diminished farther down the airway, possibly as a result of soft-tissue adaptation. Moreover, limited information is available about the long-term stability of the airway changes produced by RME. Several studies have shown that maxillary constriction may play a role in the etiology of more severe breathing disorders such as obstructive sleep apnea (OSA) in growing subjects. Early orthodontic treatment with RME is able to reduce the symptoms of OSA and improve polysomnographic variables. Finally, early orthopedic treatment with RME also is beneficial to avoid the development of facial skeletal asymmetry resulting from functional crossbites that otherwise may lead to functional and structural disorders of the stomatognathic system later in life.

## Review

### Introduction

Dentofacial orthopedic treatment, in particular rapid maxillary expansion (RME), is indicated for a wide variety of clinical conditions routinely faced by the orthodontist [1]. Frequently observed morphological problems involve an underdevelopment of the midface, which can be manifested by a constricted high-arched palate and poor transverse and/or sagittal maxillary growth [2].

Maxillary constriction can be associated with several problems that include occlusal disharmony and esthetics as well as such functional difficulties as narrowing of the pharyngeal airway, increased nasal resistance, and alterations in tongue posture, resulting in retroglossal airway narrowing and mouth breathing [3–5].

RME is an effective orthopedic procedure that has been used routinely in growing patients for over half a century. The goal of RME is to open the midpalatal suture, providing appropriate and stable maxillary width increase [6, 7]. Because of various positive side effects on the patient’s general health, the number of indications for RME has grown dramatically over the years.

Although this therapy is carried out to correct dental and skeletal maxillary transverse discrepancies, some investigators have shown that treatment outcomes also could involve increasing nasopharyngeal airway dimensions, leading to improved nasal breathing [8–10]. Constricted airways are thought to play a potential role in the pathophysiology of obstructive sleep apnea (OSA), a common health problem that if left untreated may have a deleterious impact on neurocognitive and behavioral outcomes, physical development, and cardiovascular health [11].

Traditionally, studies on the changes in upper airway dimensions have consisted of analyzing the RME posttreatment effects with two-dimensional (2D) cephalometric radiographs. Recently, the reduction in radiation dose obtained with cone beam computed tomography (CBCT) and low-dose multislice computed tomography (CT) has allowed the development of software capable of computing nasal airway volume [12].

Airway changes induced by RME treatment have been studied by means of functional examinations such as rhinomanometry (a standard diagnostic tool used to evaluate the respiratory function of the nose objectively) and acoustic rhinometry (a new technique that evaluates nasal obstruction by analyzing the reflections of a sound pulse introduced via the nostrils). These diagnostic procedures indicate a significant decrease in nasal airway resistance with consequent improvement in nasal breathing [13–15].

Polysomnography (PSG), commonly referred to as a “sleep study,” is considered the gold standard for diagnosing conditions such as obstructive sleep apnea. This diagnostic regimen provides various quantitative parameters to evaluate respiratory function such as oxygen saturation and Apnea–Hypopnea Index (AHI) [16].

Other diagnostic tools also have been introduced in orthodontics, such as electromyography (EMG), which is used to analyze the activity of the masticatory and facial muscles. EMG, a simple method of detecting and registering electric activity of muscle fibers, has been shown to have good reproducibility [17, 18].

The aim of our consensus paper was to identify and qualify the evidence of reports evaluating changes in airway dimensions and muscular function in patients treated with RME during the growth period. Studies using radiography, CBCT, magnetic resonance imaging (MRI), PSG, EMG, and ultrasound (US) were considered for this purpose. The focused questions were the following: What are the effects of RME therapy on airways, nasal cavity, and breathing function? Are these changes stable in the long term? Do children undergoing RME therapy to correct a transverse discrepancy have any long-term benefit in muscular activity?

### Search methodology

In order to identify relevant studies about the impact of RME on a child’s general health, a computerized database search was conducted using the Medline database (Medline/PubMed). The search covered the period up to March 2015. The terms used in the search were “rapid palatal or maxillary expansion” in combination with “general health,” “oral health,” “breathing,” “OSAS,” “facial musculature,” “muscle activity,” and “chewing” (Table 1). A total of 331 references were retrieved from the database search. Among them, 44 duplicate references were found.

**Table 1:** Search strategy

Search	Query	Items found
#14	(rapid palatal expansion) AND chewing	7
#13	(rapid maxillary expansion) AND chewing	8
#12	(rapid palatal expansion) AND muscle activity	3
#11	(rapid maxillary expansion) AND muscle activity	4
#10	(rapid palatal expansion) AND facial musculature	1
#9	(rapid maxillary expansion) AND facial musculature	1
#8	(rapid palatal expansion) AND OSAS	9
#7	(rapid maxillary expansion) AND OSAS	14
#6	(rapid palatal expansion) AND breathing	88
#5	(rapid maxillary expansion) AND breathing	105
#4	(rapid palatal expansion) AND oral health	27
#3	(rapid maxillary expansion) AND oral health	29
#2	(rapid palatal expansion) AND general health	4
#1	(rapid maxillary expansion) AND general health	4

For the full articles to be selected from the abstracts, they had to satisfy the following inclusion criteria: human-controlled clinical trial; growing subjects; and the use of radiography, CT, CBCT, MRI, PSG, EMG, or US to measure changes in airways, breathing, and musculature functions. The exclusion criteria were surgical expansion or other simultaneous treatment during the active expansion phase as well as systemically compromised subjects or cleft subjects.

The initial selection excluded all titles and abstracts not related to the topic or that involved any exclusion criteria. Theses, author opinion, annals, and case reports also were excluded. If the abstract contained insufficient information for a decision concerning inclusion or exclusion, the full article was obtained and reviewed before a final decision was made. Titles with no abstract available that suggested a relationship to the objectives of this review were selected to screen the full text. The reference lists of the retrieved articles also were hand searched for additional relevant publications that could have been missed in the databases.

### Airways and breathing function

Maxillary transverse deficiency is a common skeletal problem in the craniofacial region, and it is often found in children with abnormal breathing [19, 20].

According to the functional matrix theory of Moss [21, 22], only nasal breathing allows proper growth of the dentofacial complex. This theory is based on the principle that normal nasal respiratory activity influences the development of craniofacial structures, favoring their harmonious growth by adequately interacting with mastication and swallowing. Lione et al. [23] reported that prolonged mouth breathing in growing subjects influenced the development of a different palatal morphology, with a narrower and higher palatal vault compared to subjects with a nose-breathing pattern.

In recent years, there has been a growing interest in the use of three-dimensional methods of investigation such as CT and CBCT scans to evaluate the transverse effects of RME in prepubertal subjects. Podesser et al. [24], Ballanti et al. [25], Garrett et al. [26], and Palaisa et al. [27] found a mean value of expansion of the nasal cavity of 1.5 mm following RME. These findings demonstrated that the dimensional increase of the nasal cavity on the transverse plane was not limited to the anterior region, but it extended to the posterior region as well [24–27]. A systematic review by Baratieri and co-workers [7] on the skeletal effects after RME found that when the midpalatal suture is opened in growing patients, the widening of the nasal cavity is stable over the long term.

It is well accepted that the lateral displacement of the nasal cavity also is associated with an enlargement (opening) of the upper airway. Tecco et al. [28] observed that the RME group with a mean age of 8 years had a significant increase in nasopharyngeal airway dimension (5.3 mm) compared with a matched control group (1.2 mm). This airway improvement occurred 6 months after RME and remained stable at a 12-month follow-up examination. The clinical significance of these findings is that RME causes decreased nasal airway resistance, which results in a reduction of head elevation and suggesting improvement in nasal breathing [28]. In growing patients with maxillary constriction treated with RME, Ribeiro et al. [29], Smith et al. [30], Chang et al. [31], Zeng and Gao [32], and Hakan and Palomo [33] observed dimensional changes of the upper airway as assessed by means of CBCT. An increased cross-sectional area immediately posterior to the hard palate was found. The effect of RME on the upper airway was limited and local; it diminished farther down the airway, possibly as a result of soft-tissue adaptation [29–33].

The examination of the upper airway plays an important role in the evaluation of the growth and general health of subjects with breathing disorders. Because of the great complexity of airway anatomy and function, several measurement methods have been proposed. These methods can complement each other in the assessment of changes in breathing function after RME [2, 34].

De Felippe et al. [19], by means of 3D morphometric analysis and of acoustic rhinometry evaluation under basal conditions, found an increase in the minimal cross-sectional area of the nasal cavity, concomitant with a 34 % reduction in nasal airway resistance immediately after RME. These authors also observed stability of the results in a long-term follow-up (60 months after RME), with values comparable to those of subjects with normal nasal breathing conditions [19].

Enoki et al. [35] evaluated the effects of RME on the nasal cavity in 29 children and compared computed rhinomanometric values before, immediately after, and 90 days after RME. Their results showed no significant difference for the minimal cross-sectional airway at the level of the nasal valve. Nevertheless, despite the absence of minimal cross-sectional airway changes, the computed rhinomanometry found a progressive decrease in the inspiration and expiration resistances, reaching statistical difference from before RME to 90 days after RME. These findings indicate that the benefits of RME might be a modest functional improvement based on bony expansion rather than a mucosal dimensional change [35].

Iwasaki et al. [36] used CBCT and computational fluid dynamics to estimate the effects of RME on nasal airflow function (pressure and velocity) in 22 subjects with a mean age of 9.10 years without morphologic obstruction. In 18 patients undergoing expansion, the pressure and velocity of nasal ventilation after RME resulted significantly lower than before treatment indicating an improvement in nasal breathing [36].

Fastuca et al. [37, 38] evaluated changes in airway volumes and respiratory performance in 15 patients with a mean age of 7.5 years undergoing RME to determine whether any correlations exist between the morphological and respiratory functional modifications. On CBCT, the airway regions were segmented and the volumes were computed to detect variations after the removal of the maxillary expander 12 months later. The multiple logistic regressions showed that the more a subject presented with a reduced nasal volume in the middle and lower compartments, the more he or she would benefit from RME in terms of improved oxygen saturation.

The Apnea–Hypopnea Index (AHI) can be used to indicate the severity of sleep apnea. Evaluating AHI as a secondary outcome, Fastuca et al. [38] found an improvement in the index with a reduction in apneic events of 4.2 per hour. Not only the upper and nasal airways but also the middle and lower airway compartments underwent significant volume increases. Such increases were greater for the nasal cavity and slightly lower for the middle and lower compartments [38].

### Obstructive sleep apnea

Several studies have shown that maxillary constriction may play a role in the etiology of more severe breathing disorders such as obstructive sleep apnea (OSA) in growing subjects [3, 11]. OSA is a condition characterized by the episodic cessation of breathing during sleep. An examination of the causes of apnea has produced several classifications for this condition. Apnea secondary to sleep-induced obstruction of the upper airway and combined with simultaneous respiratory efforts is the most common type; it has been classified as obstructive sleep apnea syndrome (OSAS).

OSAS results in oxygen desaturation and arousal from sleep, thus bringing several signs and symptoms related to oxygen desaturation and sleep fragmentation [39]. Impaired sleep quality leads to excessive daytime sleepiness, deterioration of memory and judgment, altered personality, and reduced concentration. Growth hormone is produced during slow-wave sleep, and its secretion may be interrupted by fragmented sleep. The increase in effort for breathing to overcome obstruction and its consequent calorie demand are a further mechanism by which OSA impacts growth. Moreover, the reduced blood oxygen saturation may give rise to hypertension, cardiac arrhythmia, nocturnal angina, and myocardial ischemia [40].

Under physiological conditions, the nose accounts for 50 % of respiratory resistance. Nasal obstruction related to both anatomical–structural and functional causes is an important risk factor for OSAS. Reducing nasal resistance, therefore, is one of the main objectives of therapy with RME. By widening the palate, RME increases the volume of the nasal and buccal cavities, thus helping to reduce nasal resistance [41, 42].

Several investigators have analyzed growing patients from 6 to 13 years of age with oral breathing, snoring, and nighttime apnea history treated with RME. These studies have demonstrated that at the end of orthopedic treatment, RME was effective in children affected by OSAS without any other obvious obstruction of the upper respiratory airways. RME therapy widened the nasal fossae and released the septum, thus restoring normal nasal airflow with the disappearance of obstructive sleep disordered breathing [43–45].

Marino et al. [46] evaluated the effects of RME in a group of 15 OSAS preschool children after a mean period of 1.5 years. These investigators found that at the end of the overall observation period, approximately half of the patients demonstrated an improvement of the Respiratory Disturbance Index (RDI). The RDI reflects the average number of apneas plus hypopneas observed per hour of sleep; it usually is obtained by identifying and manually counting each respiratory disturbance with subsequent division of the sum by the number of hours slept [47].

The use of RME to enhance respiratory function was more effective in OSAS children with bimaxillary retrusion. Maxillary retrusion has been suggested to constrict the upper airway including the nasal cavity and velopharynx and affect airway size or ventilation [46].

Over a 36-month follow-up period, Villa et al. [48] observed subjects between 4 and 11 years old with clinical signs of malocclusion (all presented with a high, narrow palate associated with deep bite, retrusive bite, or crossbite). The subjects also had signs and symptoms of OSA, including habitual snoring, apneas, and restless sleep witnessed by parents. They were characterized by an obstructive apnea/hypopnea index >1 proven by laboratory polysomnography; the parents of these children had refused adenotonsillectomy as a treatment alternative. Early orthodontic treatment with an RME in these children resulted in reduced symptoms of OSAS and improved polysomnographic variables. In 10 of the 14 patients who completed treatment (71 %), the symptoms of OSAS regressed, and in 79 % of treated patients, the AHI decreased significantly. In all patients, the oral breathing disappeared after orthopedic therapy because of the enlarged space available for the adenoids and tonsils [48].

Pirelli et al. [49] evaluated the long-term efficacy of RME in 23 individuals followed up for 12 years from an initial sample of 31 children.At baseline the subjects presented with a mean age of 8.7 years, OSA diagnosis, maxillary contraction, and absence of enlarged adenotonsils[49].

Guilleminault et al. [45] conducted a clinical trial of 31 children with a mean age of 6.5 years who had been diagnosed with OSAS based on clinical signs and symptoms and who had undergone a sleep study. They were randomized into two groups: group 1 received adenotonsillectomy followed by orthodontics, while group 2 received orthodontics followed by surgery. The authors reported that there was no significant difference between the group beginning with orthodontic treatment and the one beginning with surgical treatment after the first intervention. Most children, however, needed both treatments to have complete resolution of their symptoms and normalization of PSG [45].

### Muscle activity

It is well accepted that occlusal factors may influence and cause functional disturbances of the masticatory system [50]. In particular, maxillary constriction and functional unilateral posterior crossbite have been shown to be associated with temporomandibular disorders and altered muscle function. The diagnosis of “functional crossbite” is based on the presence of a mandibular shift from centric relation to an asymmetric intercuspal position. Patients with functional crossbites have symmetric mandibles that merely are positioned laterally [51].

Several investigators have demonstrated through tomographic evaluation asymmetric condylar position in centric occlusion in children with unilateral posterior crossbite, due to an anterior and inferior position of the condyle within the glenoid fossa on the non-crossbite side compared with that of the crossbite side [52–54]. During the growth period, continuous condylar displacement in the glenoid fossa induces differential growth of the condyles and of the mandibular ramus, leading to skeletal asymmetry [54].

The functional mandibular shift also is related with postural adjustments. EMG studies have shown that the activity of the temporal and masseter muscles in functional crossbite patients is disturbed [55]. The position of the mandible at rest is held by the viscoelastic properties of muscles and tendons that counterbalance the force of gravity. At rest, EMG activity should be minimal or completely absent [56].

De Rossi et al. [5] found that children with crossbite have EMG activity for all the masticatory muscles in rest position. This observation is due most likely to an increase in basal tonus in the stomatognathic system [5]. Children with functional crossbite have greater resting activity of the anterior temporalis muscle on the non-crossbite side and of the posterior temporalis muscle on the crossbite side when compared with healthy subjects [57].

The asymmetrical mandibular position induces different development of the elevator muscles on each side of the jaws leading to a thinner masseter muscle on the crossbite side, which already can be seen in the early mixed dentition [58]. Egermak-Eriksson [59] observed that individuals with crossbite more often chew unilaterally than those that had no crossbite. Ingervall and Thilander [60] found in children aged 8–12 years an abnormal chewing pattern (i.e., reverse sequencing), which was interpreted as an adaptation to avoid cuspal interferences. Chewing cycles are similar to the pattern of patients with temporomandibular dysfunctions and different from the pattern of the subjects with normal bites. After the elimination of crossbites, cycles generally become more regular and symmetrical, comparable to those of patients with normal occlusion [60].

Thus, crossbite is a morphological malocclusion that seems to predispose to functional disturbances, which might be taken as indicating treatment as early as possible [58]. RME is considered the treatment of choice for functional crossbite because it eliminates the lateral functional mandibular shift, preventing the development of skeletal asymmetry and of muscle function disturbances [56, 59].

## Conclusions

RME is an effective orthopedic procedure used to treat structural and functional problems in the midface. Such treatment has been shown to have positive side effects on the general health of the patient. The widening of the nasal cavity base, found after midpalatal suture opening in growing patients, allows for the reduction in nasal airway resistance and improvement in the respiratory pattern. The significant improvement of the volume of the upper airway that remains stable in the long term suggests a fundamental role of dentofacial orthopedics in the treatment of not only maxillary constriction but also of constrictions of the nasopharyngeal spaces associated with oral breathing, snoring, and obstructive sleep apnea syndrome during childhood. The effects of RME on the upper airway, however, have been described as limited and local, and they diminished farther down the airway, possibly as a result of soft-tissue adaptation. Moreover, very limited information is available about the long-term stability of the airway changes produced by RME. Several studies have shown that RME can be beneficial in the treatment of maxillary constriction associated with more severe breathing disorders such as obstructive sleep apnea (OSA) in growing subjects.

Early orthopedic treatment with RME also is beneficial in avoiding the development of facial skeletal asymmetry that may lead to both functional and structural imbalances in growing patients. If left uncorrected, such a functional shift may lead to the development of temporomandibular disorders and other related conditions later in life.
